# LncRNA AWPPH as a prognostic predictor in human cancers in Chinese population: evidence from meta-analysis

**DOI:** 10.1042/BSR20210012

**Published:** 2021-06-08

**Authors:** Yongfeng Li, Xinmiao Rui, Daobao Chen, Haojun Xuan, Hongjian Yang, Xuli Meng

**Affiliations:** 1 Department of Breast Surgery, Zhejiang Provincial People’s Hospital, Affiliated People's Hospital, Hangzhou Medical college, Hangzhou, Zhejiang 310022, P.R. China; 2 Department of Breast Surgery, Institute of Cancer Research and Basic Medical Sciences of Chinese Academy of Sciences (Zhejiang Cancer Hospital), Zhejiang, China; 3 Second Clinical Medical College, Zhejiang Chinese Medical University, Hangzhou, Zhejiang, P.R. China

**Keywords:** AWPPH, long noncoding RNA, meta-analysis, prognosis

## Abstract

Background: Long non-coding RNA associated with poor prognosis of hepatocellular carcinoma (AWPPH) is dysregulated in a variety of human cancers. However, the prognostic value of AWPPH in various cancers remains unclear.

Methods: Comprehensive literature search was performed in PubMed, Web of Science, CNKI and Wangfang databases, and eligible studies were obtained according to the inclusion and exclusion criteria. The pooled hazard ratios (HRs) and odds ratios (ORs) were applied to assess the clinical value of AWPPH expression for overall survival (OS) and clinicopathological features.

Results: A total of 19 articles including 1699 cancer patients were included in the study. The pooled results demonstrated that evaluated AWPPH expression was positively related to a poorer overall survival of patients with cancers (HR = 1.79, 95%CI: 1.44–2.14, *P*<0.001). Subgroup analysis revealed that tumor type and sample size affect the predictive value of AWPPH on OS, whereas cut-off value and HR estimation method have no impact on it. In addition, the pooled data also showed that AWPPH was positively linked to advanced TNM stage (OR = 2.50, 95%CI: 1.94–3.22, *P*<0.001), bigger tumor size (OR = 2.64, 95%CI: 1.47–4.73, *P*=0.001), macro-vascular invasion (OR = 2.08, 95%CI: 1.04–4.16, *P*=0.04) and lymph node metastasis (OR = 2.68, 95%CI: 1.82–3.96, *P*<0.001). Moreover, the results of the trim and fill analysis confirmed the reliability of our finding.

Conclusions: Up-regulation of AWPPH was associated with advanced TNM stage, bigger tumor size, worse lymph node metastasis, macro-vascular invasion and shorter overall survival, suggesting that AWPPH may serve as a biomarker for prognosis and clinicopathological characteristics in human cancers among the Chinese population.

## Background

Cancer is a major public health problem worldwide, and it has been the leading cause of death in China since 2010 [1]. Cancer is a highly complex disease involving numerous molecular changes, including chromosomal translocations, deletions and amplification, epigenetic alterations and genetic mutations [2–4], which make it more difficult to be cured than ordinary diseases. Although great advances have been achieved in diagnoses and treatments, the clinical prognosis remains undesirable in most cancer patients. Therefore, the exploration of effective molecular biomarkers that can be used to guide clinical prevention, treatment and prognosis prediction of cancer is becoming imminent.

LncRNA is a typical kind of non-coding RNA without meaningful open reading frame, which also possesses many significant functions and plays important roles in tumorigenesis and tumor progression. Most lncRNA transcripts involved in the epigenetic, transcriptional, and posttranscriptional regulation of cancer cells [5]. Furthermore, a variety of lncRNA could function as enhancers [6], splicing regulators [7], as well as chromatin remodelers [8]. Notably, accumulating evidence demonstrated that dysregulated lncRNA occurred in a broad spectrum of human cancers [9,10]. These cancer-related lncRNAs have been proved to participate in cancer initiation and progression, which may have potential value as clinical biomarkers and therapeutic targets. Recently, the long non-coding RNA associated with poor prognosis of hepatocellular carcinoma (AWPPH) attracted increasing attention.

AWPPH, also well-known as AK001796, MIR4435-2HG, LINC00978 and other names, was localized at 2q13 and found to be dysregulated in many human cancers. Growing evidence showed that AWPPH was associated with tumorigenesis and prognostic outcome [11–13]. However, abundant studies reported the prognostic value of AWPPH for human cancers were constrained by sample size and discrete outcome so far. Consequently, we performed this systematic review and meta-analysis on the basis of eligible retrospective studies to investigate the potential prognostic value of AWPPH for cancer patients.

## Methods

### Literature collection

This meta-analysis was performed in accordance with the PRISMA 2009 guidelines (Supplementary S1) [14]. We performed literature search using PubMed, Web of Science, CNKI and Wangfang database for eligible studies which reported the relationship between lncRNA AWPPH and prognosis of human cancers before October 5, 2020. Search terms used as follows: (‘carcinoma’ OR ‘cancer’ OR ‘tumor’ OR ‘neoplasm’) AND (‘prognosis’ OR ‘outcome’ OR ‘diagnosis’ OR ‘survival’) AND (‘AWPPH’ OR ‘LINC00978’ OR ‘MIR4435-1HG’ OR ‘MORRBID’ OR ‘AGD2’ OR ‘MIR4435-12HG’ OR ‘AK001796’ OR ‘MIR4435-2HG’). The reference lists of primary publications were also manually searched to obtain potential eligible studies. There is no requirement for patient consent or ethical approval due to all the analyses were conducted on the basis of the prior published researches.

### Inclusion and exclusion criteria

Eligible studies should meet the following inclusion criteria : (1) Studies evaluated the association between AWPPH and cancer patient samples; (2) Available prognosis outcomes or clinicopathologic features data; (3) sufficient information to obtain hazard ratio (HR) or odds ratio (OR) with 95% confidence interval (95% CI). The following articles were excluded from the study: (1) reviews, letters or case reports; (2) non-human studies; (3) duplicated publication; (4) studies with insufficient data for HR/OR/95%CI extraction.

### Data extraction and quality assessment

Eligible articles were reviewed by two reviewers (Li and Rui) independently according to the inclusion and exclusion criteria. Disagreement was resolved during a consensus with a third reviewer (Chen). The essential information was screened and extracted from each eligible study, including the name of first author, year of publication, origin country, cancer type, sample size, detection method of AWPPH, HR and corresponding 95%CI for OS, as well as clinicopathological features. The HRs with 95%CIs were obtained directly from studies which performed the multivariate analysis, and the Kaplan–Meier curves were used for the extraction of the survival information if the 95% CIs and HRs have not been directly reported from the researches according to the method described in the previous publication [15]. The Newcastle–Ottawa Scale (NOS) was applied to evaluate the quality of the included study. The NOS scores ranged from 0 to 9 and studies with a NOS score >6 were considered to be high quality.

### Statistical analysis

The present meta-analysis was performed with STATA SE 15.0 (Stata Corporation). HR and corresponding 95%CI for OS were applied to determine the pooled effect for clinical outcomes, and the odds ratio (OR) with 95%CI were used to evaluate the correlation between LncRNA AWPPH and clinicopathological parameters. Statistical heterogeneity was assessed using the *I*^2^ test as well as the chi-based *Q*-test, to determine heterogeneity between several studies. Heterogeneity was considered as statistically significant with *I*^2^ < 50%. The fixed-effect model was used if heterogeneity exists (*I*^2^ > 50% and *P*<0.05), otherwise, the random-effect model was applied. Publication bias was assessed using Begg’s funnel plot and Egger’s regression test. The sensitivity analysis was used to check the stability of the combined results and to determine the source of any heterogeneity. The P-value <0.05 was considered to be statistically significant.

## Results

### Summary of eligible studies

**Figure 1:**
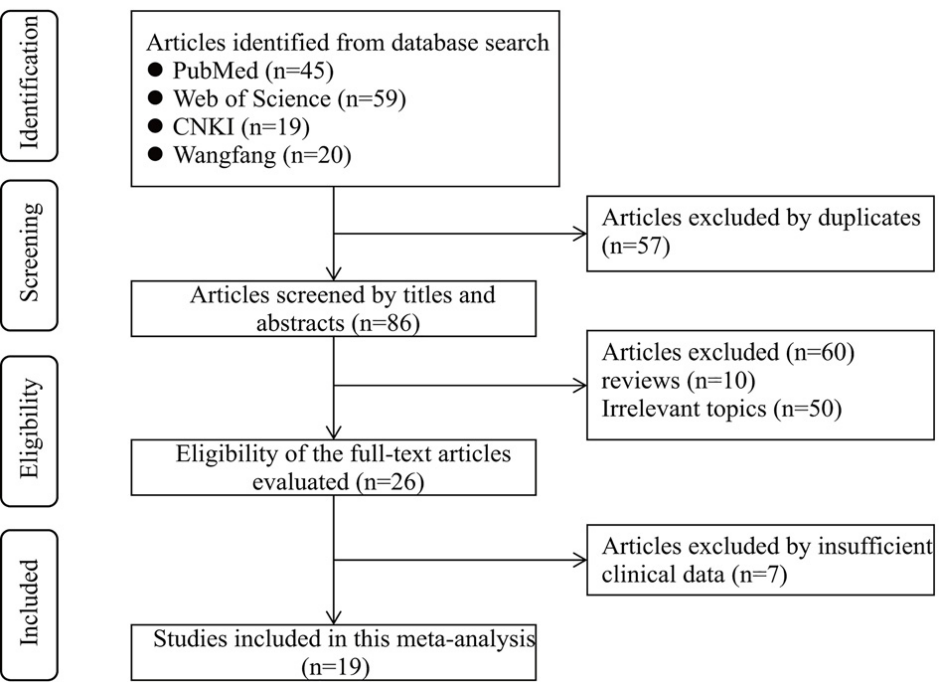
Flow chart of literature search

As shown in Figure 1, a total of 143 potentially relevant articles were obtained from the first attempt to search by using the keywords. There are 57 duplicate articles and 60 irrelevant articles excluded after screening the titles and abstracts. Finally, 7 studies with insufficient data were excluded and the remain 19 studies were included in the subsequent meta-analysis. The main characteristics of the included 19 studies have been summarized in Table 1. A total of 1699 patients from 19 studies between 2016 and 2020 were included [11–13,16–31]. The respective sample sizes ranged from 36 to 195 patients. 19 studies had addressed 12 different types of cancer: including hepatocellular carcinoma (HCC, *n*=3), colorectal adenocarcinoma (CRC, *n*=3), ovarian carcinoma (OC, *n*=2), triple-negative breast cancer (TNBC, *n*=1), non-small cell lung cancer (NSCLC, *n*=2), osteosarcoma (*n*=1), cervical cancer (CC, *n*=1), oral squamous cell carcinom (OSCC, *n*=1), clear cell renal cell carcinoma (CCRCC, *n*=1), GC (*n*=1), prostate carcinoma (PC, *n*=1), breast cancer (BC, *n*=1), esophageal squamous cell carcinoma (ESCC, *n*=1). Clinical outcomes were recorded including 19 studies for overall survival (OS), 3 for recurrence-free survival (RFS), 1 for progression-free survival (PFS), and 1 for disease-free survival (DFS). HRs with corresponding 95% CIs were obtained from the original data in 4 studies, and calculated from Kaplan–Meier curves for the rest 15 studies. In addition, for the quality assessment, the Newcastle–Ottawa Scale (NOS) score of individual cohort studies was ranged from 6 to 8, which indicated that the methodological quality of included studies was medium or high. The clinicopathological features of the included studies were summarized in Table 2.

**Table 1:** Characteristics of the included eligible studies

Author	Year	Country	Tumor	Sample size	Cut-off value	Detection method	Outcomes	HR estimation method	HR (95%CI)	NOS
Zhao, X.D.	2017	China	HCC	88	Median	qRT-PCR	OS/RFS	U/M	OS: 3.509 (1.574–7.820) RFS: 2.579 (1.425–4.668)	8
Liu, C.C.	2018	China	CRC	86	Median	qRT-PCR	OS	Indirectly	1.51 (0.74,3.07)	8
Yu, G.Y.	2019	China	OC	58	Median	qRT-PCR	OS	Indirectly	2.05 (1.01,4.14)	7
Wang, K.N.	2018	China	TNBC	68	Median	qRT-PCR	OS	Indirectly	1.79 (0.90,3.59)	8
Song, Z.	2018	China	NSCLC	88	Median	qRT-PCR	OS	Indirectly	Tissue: 1.78 (0.99,3.20) Serum: 1.66 (0.91,3.05)	8
Li, H.	2019	China	Osteosarcoma	36	Median	qRT-PCR	OS/RFS	Indirectly	OS: 0.53 (0.14,2.00) RFS: 0.56 (0.14,2.29)	7
Wu, D.	2020	China	NSCLC	56	Median	qRT-PCR	OS	Indirectly	2.861 (1.439–5.686)	8
Chen, X.H.	2020	China	CC	75	Mean	qRT-PCR	OS	U/M	2.104 (1.221–3.626)	8
Ma, X.D.	2020	China	OSCC	82	Mean	qRT-PCR	OS	Indirectly	7.24 (1.58,33.10)	8
Dong, X.H.	2020	China	CRC	90	Median	qRT-PCR	OS	Indirectly	1.30 (0.44,3.80)	8
Ho, J.Q.	2020	China	CCRCC	118	Median	qRT-PCR	OS/RFS	Indirectly	OS: 2.98 (0.52,17.17) RFS: 2.17 (0.65,7.18)	8
Bu, J.Y.	2018	China	GC	150	Median	qRT-PCR	OS	Indirectly	1.97 (1.24,3.14)	7
Zhu, L.J.	2020	China	OC	42	Median	qRT-PCR	OS	Indirectly	1.85 (0.65,5.26)	8
Zhang, H.	2019	China	PC	68	Mean	qRT-PCR	OS	Indirectly	1.83 (0.83,4.03)	6
Shen, M.Y.	2020	China	CRC	102	Mean	qRT-PCR	OS/PFS	Indirectly	OS: 2.57 (0.98,6.74) PFS: 3.18 (1.20,8.39)	7
Zhang, Q.	2020	China	HCC	49	Mean	qRT-PCR	OS	Indirectly	1.96 (0.66,5.84)	7
Deng, L.L.	2016	China	BC	195	Mean	qRT-PCR	OS	U/M	2.27 (1.237,4.165)	8
Han, Q.L.	2019	China	HCC	73	Median	qRT-PCR	OS	Indirectly	2.02 (1.04,3.92)	8
Zong, M.Z.	2019	China	ESCC	175	Median	qRT-PCR	OS/DFS	U/M	OS: 3.347 (1.423,5.457) DFS: 3.568 (1.537,5.778)	8

Abbreviations: BC, breast cancer; CC, cervical cancer; CCRCC, clear cell renal cell carcinoma; CRC, colorectal adenocarcinoma; DFS, disease-free survival; ESCC, esophageal squamous cell carcinoma; GC, gastric cancer; HCC, hepatocellular carcinoma; NOS, Newcastle-Ottawa Scale; NSCLC, non-small cell lung cancer; OC, ovarian carcinoma; OS, overall survival; OSCC, oral squamous cell carcinoma; PC, prostate carcinoma; PFS, progression-free survival; RFS, recurrence-free survival; TNBC, triple-negative breast cancer; U/M, univariate/multivariate analysis.

**Table 2:** The clinicopathological features of the included studies

Author	Year	AWPPH expression		TNM		Tumor size		Macro-vascular invasion		Lymph node metastasis	
		high	low	>I stage in HG	>I stage in LG	>50 in HG	>50 in LG	Yes in HG	YES in LG	Yes in HG	YES in LG
Zhang, Q.	2020	26	23	16	16	14	14	10	7		
Zhu, L.J.	2020	21	21			11	7			13	6
Ma, X.D.	2020	20	62	12	22					9	8
Dong, X.H.	2020	45	45	24	10	29	17			32	30
Han, Q.L.	2019	37	36	19	8	25	9				
Shen, M.Y.	2020	55	47	30	15	40	15			38	14
Zhao, X.D.	2017	44	44	33	24	26	24	28	18		
Zong, M.Z.	2019	87	88	39	24					35	22
Wang, K.N.	2018	34	34	26	14	14	5				
Ho, J.Q.	2020	59	59	32	11						
Deng, L.L.	2016	49	146	18	37						
Zhang, H.	2019	31	37	17	21						
Li, H.	2019	19	17	15	6						
Yu, G.Y.	2019	29	29								
Song, Z.	2018	44	44								

Note: HG represented the group with high AWPPH expression, LG represented the group with low AWPPH expression.

### Prognostic value of AWPPH

**Figure 2:**
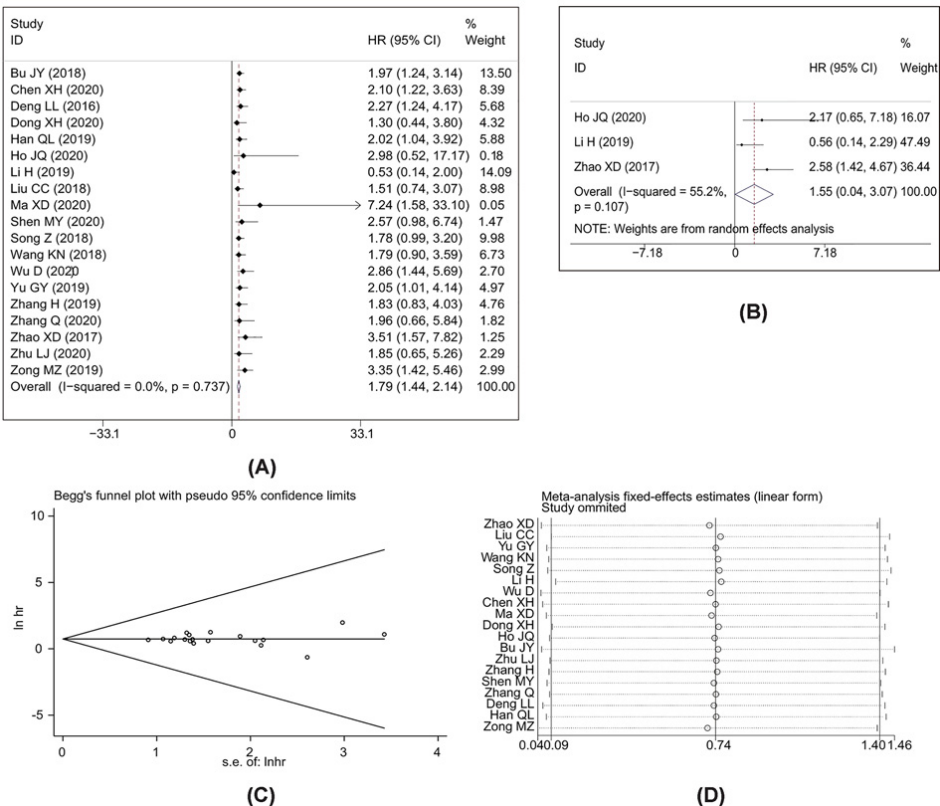
Meta-analysis of the association between AWPPH expression and prognosis index (**A** and **B**) Forest plot and of studies evaluating the association between AWPPH expression and OS and RFS. (**C**) Begg’s publication bias plots of OS, and (**D**) sensitivity analysis for OS.

**Figure 3:**
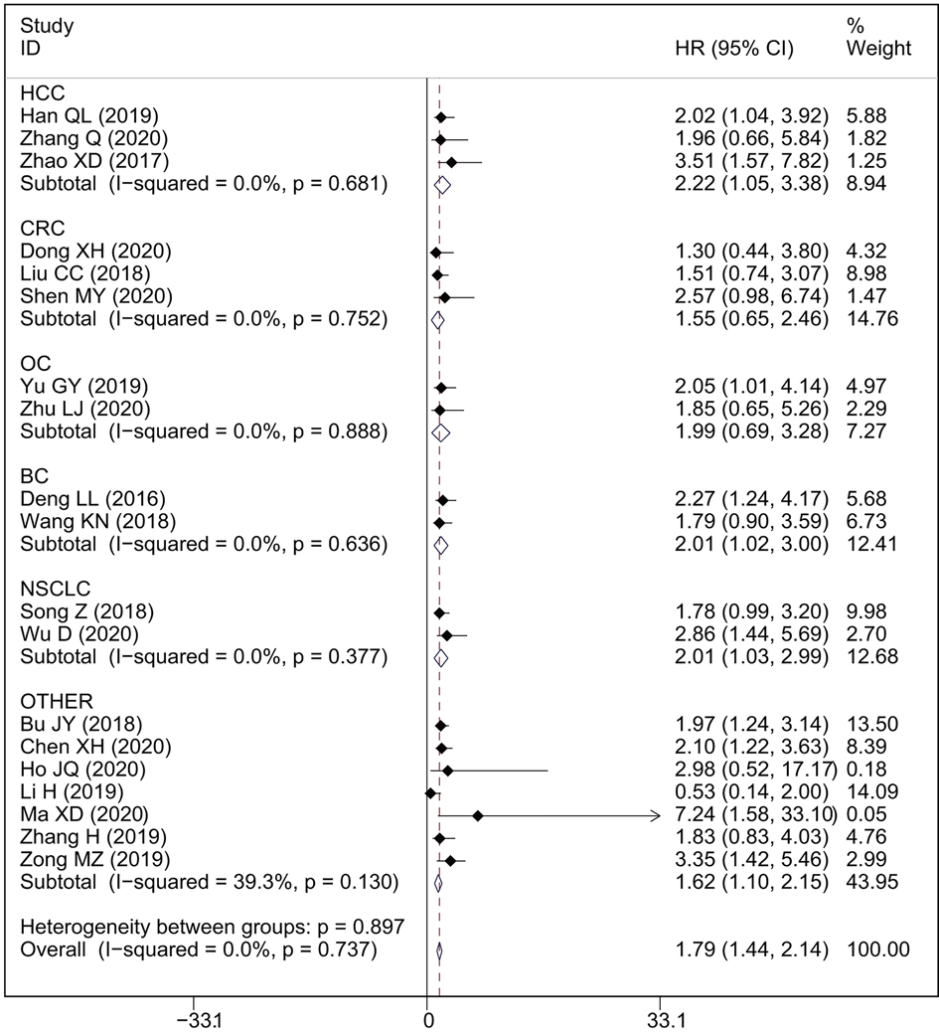
Forest plots of subgroup analysis for the HRs of OS by tumor type

**Figure 4:**
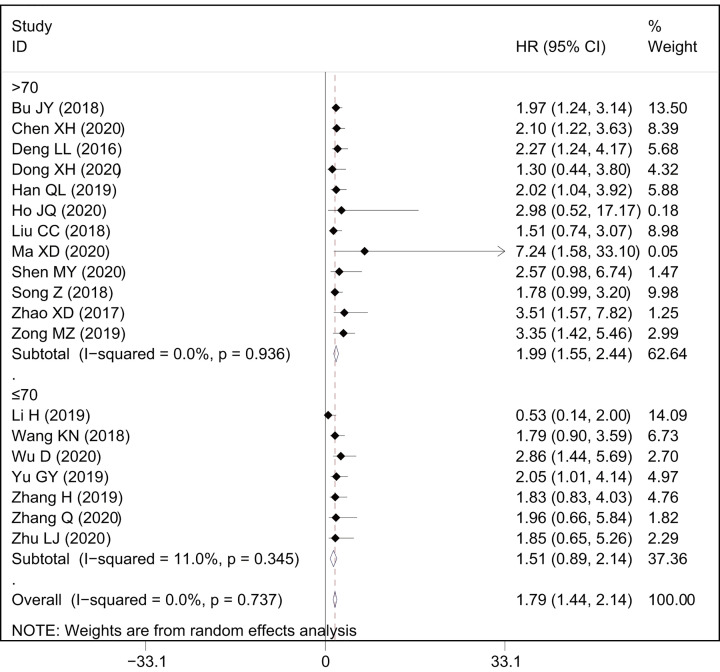
Forest plots of subgroup analysis for the HRs of OS by sample size

**Figure 5:**
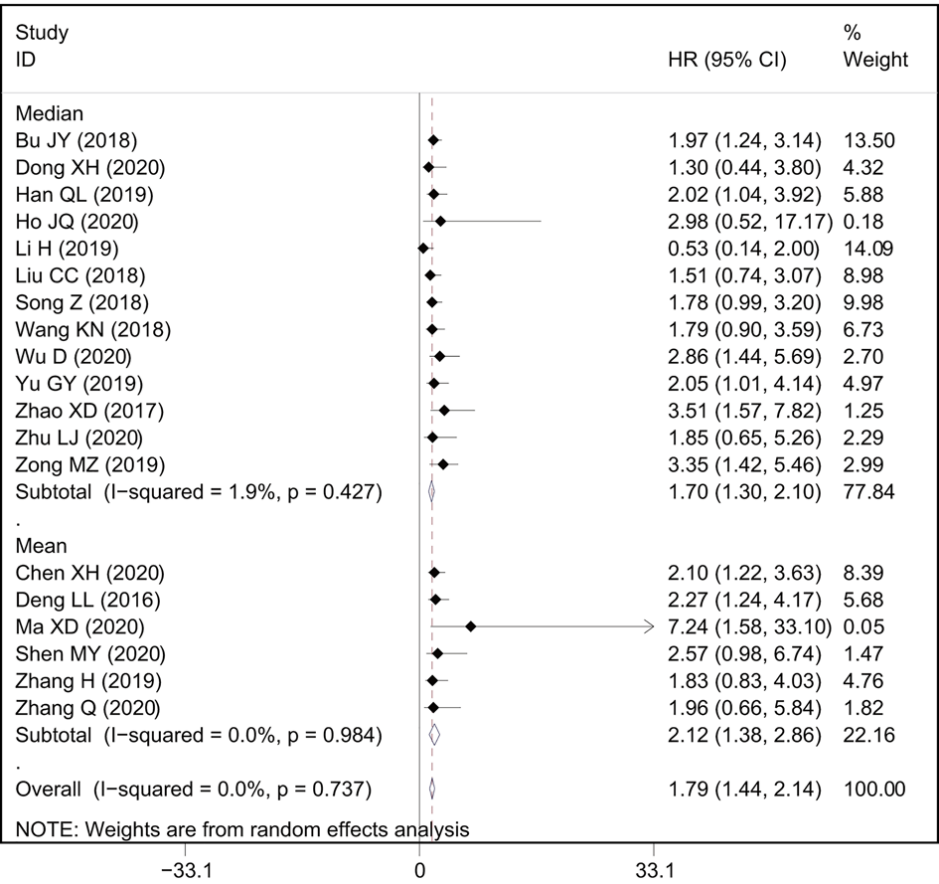
Forest plots of subgroup analysis for the HRs of OS by cut-off value

**Figure 6:**
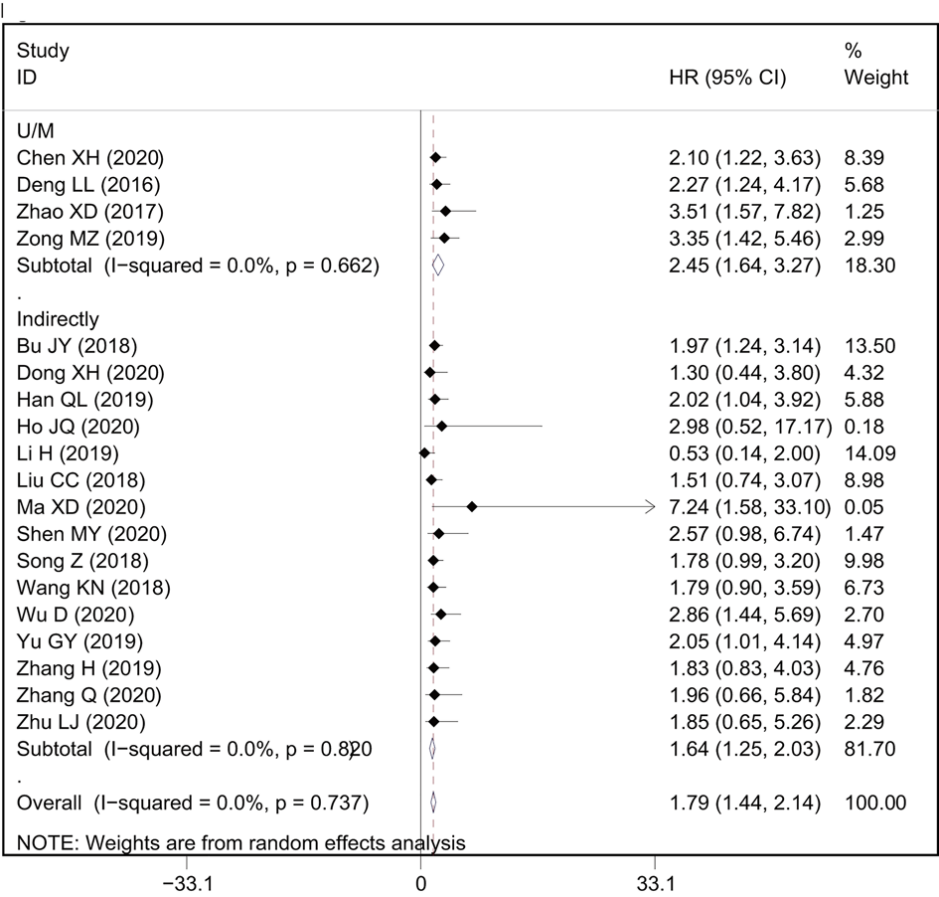
Forest plots of subgroup analysis for the HRs of OS by HR estimation method

A total of 19 studies with 1699 patients reported the relationship between OS and AWPPH in human cancers. As shown in Figure 2A, a significant correlation was observed between elevated AWPPH expression and poor OS (HR = 1.79, 95%CI: 1.44–2.14, *P*<0.001) with non-significant heterogeneity (*I*^2^ = 0%, *P*=0.737). Furthermore, subgroup analysis across several different variables, including cancer type, sample size, HR estimation method, and cut-off value, were further performed to explore the association between HRs and OS. The results showed that cancer type and sample size influence the prognostic value of AWPPH on OS (Figures 3 and 4), whereas the HE estimation methods and cut-off value have no impact on it (Figures 5 and 6). There was a negatively relationship between AWPPH expression and OS in the patients HCC (HR = 2.22, 95%CI: 1.05–3.38), NSCLC (HR = 2.01, 95%CI: 1.03–2.99), BC (HR = 2.01, 95%CI: 1.02–3.00), and other cancers (HR = 1.62, 95%CI: 1.10–2.15) (Figure 3). Moreover, the effect of AWPPH over-expression on predicting short OS occurred in the studies with sample size >70 (HR = 1.99, 95%CI: 1.55–2.44) (Figure 4).

### Association between AWPPH and clinicopathological features

**Figure 7:**
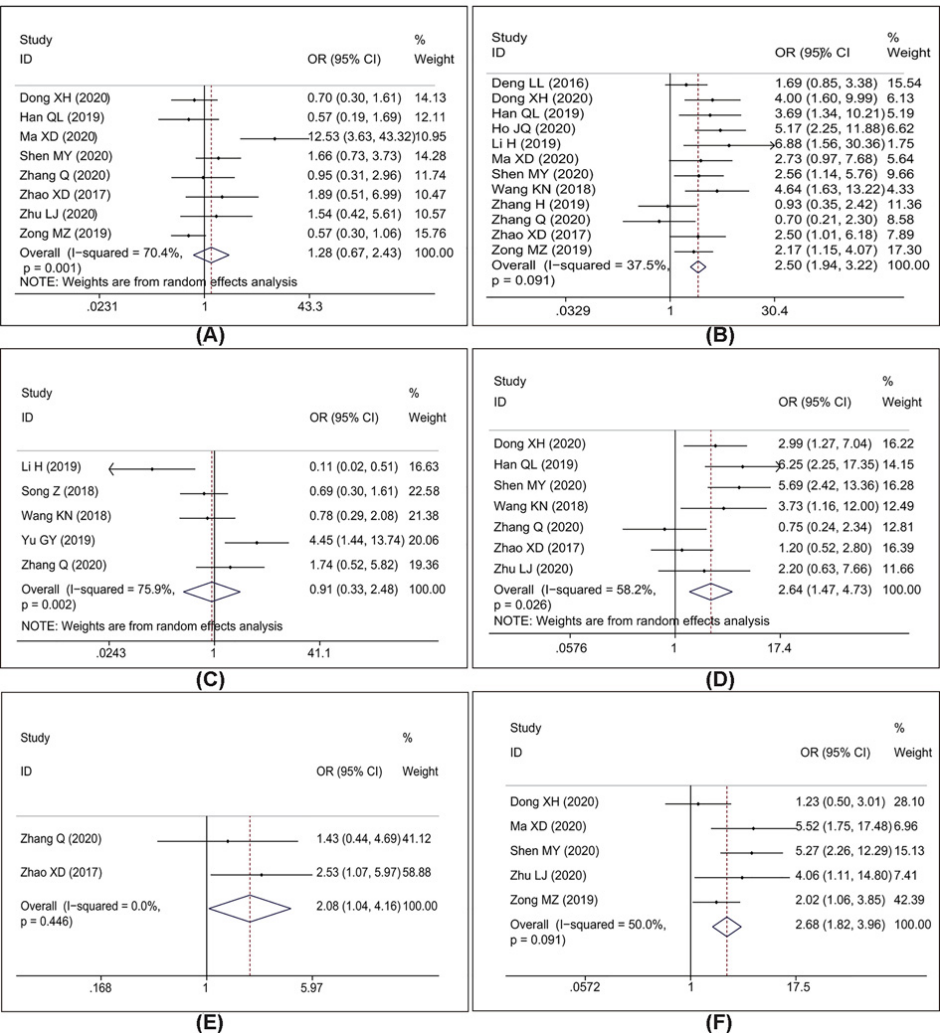
Meta-analysis for the association between AWPPH expression with clinicopathological parameters The investigated clinicopathological parameters are: (**A**) differentiation status, (**B**) TNM stage, (**C**) distant metastasis, (**D**) tumor size, (**E**) macro-vascular invasion and (**F**) lymph node metastasis.

The correlation between AWPPH expression and clinicopathological characteristics were examined with OR analysis in 15 studies with 1332 cancer patients (Figure 7 and Table 3). About 12 studies with 1143 patients were included to analysis the link between AWPPH and TNM stage, and the pooled data found an obvious association between AWPPH overexpression and advanced TNM stage (OR = 2.50, 95%CI: 1.94–3.22, *P*<0.001) (Figure 7B). The results also showed that over-expression of AWPPH predicts larger tumor size (OR = 2.64, 95%CI:1.47–4.73, *P*=0.001, Figure 7D). In addition, 2 studies with 137 patients were included to analyze the link between AWPPH and macro-vascular invasion, the results revealed an obvious association between AWPPH expression and MVI (OR = 2.08, 95%CI: 1.04–4.16, *P*=0.039, Figure 7E). As shown in Figure 7F, 491 cancer patients from 5 studies were included to evaluate the correlation between AWPPH and LNM, and the results indicated that the patients with elevated AWPPH expression were more susceptibility to develop LNM (OR = 2.68, 95%CI: 1.82–3.96, *P*<0.001).

**Table 3:** The *P*-values obtained from either the fixed or random model for the risk association analyses

Risk factors	Models	*P* value
Differentiation	Random effect	0.45
Distant metastasis	Random effect	0.854
Lymph node metastasis	Fixed model	<0.001
Macro-vascular invasion	Fixed model	0.039
TNM stage	Fixed model	<0.001
Tumor size	Random effect	0.001

### Publication bias and sensitivity analysis

Begg’s funnel plot and Egger’s linear regression tests were introduced to evaluate potential publication bias in our present meta-analysis. In the analysis of evaluating the association between AWPPH expression and OS, visual inspection of the Begg’s funnel plot did not reveal asymmetry (Figure 2C), and Egger’s test also suggested the absence of publication bias (*t* = 0.06, *P*=0.953). Sensitivity analyses were performed to evaluate whether individual study influenced pooled HRs by excluding one study by turns. The results showed that the pooled HR was not significantly changed after removing each study, suggested that the results were stable (Figure 2D).

## Discussion

Evidence from multiple publications demonstrated that lncRNA AWPPH is closely associated with cancers. AWPPH was first discovered in breast caner as the name of LINC00978 [26]. In breast cancer patients, the expression of AWPPH was negatively associated with hormone receptor status, and high AWPPH expression predicted poor DFS. In recent years, It has been shown from prior studies that AWPPH serves as a dysregulated oncogene in several cancers, such as GC [24], CRC [32] and NSCLC [30]. AWPPH can promote cell proliferation, migration and invasion in a variety of human cancers, and played an crucial role in tumor progression, metastasis and prognosis. However, a persuasive support of the AWPPH in clinical practice is still controversial. In order to combine previous research results about AWPPH and cancers to arrive at a summary conclusion, a comprehensive study was carried out.

In this meta-analysis, we pooled data from a total of 19 retrospective eligible studies with 1699 cancer patients to systematically explore the relationship between AWPPH and prognosis. We found that elevated AWPPH expression was an unfavorable prognostic factor in multiple cancer patients. Furthermore, the results also demonstrated that high AWPPH expression level was positively related to advanced TNM stage, higher risk of LNM and MVI, and bigger tumor size.

**Figure 8:**
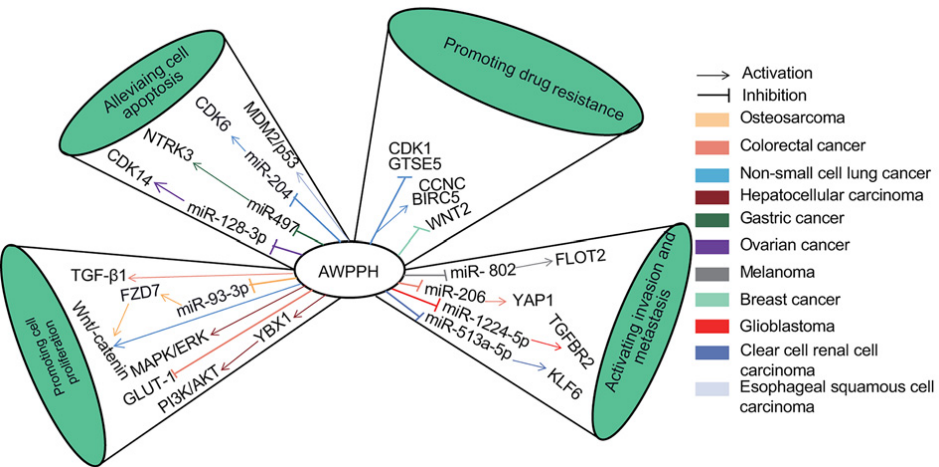
Schematic diagrams of various molecules and signaling pathways associated with AWPPH in human cancers

The exact mechanisms underlying the association between aberrant AWPPH expression and poor clinical prognosis remains elusive. The molecular mechanism of AWPPH in various cancers from prior studies were illustrated in Figure 8. Previous study reported that AWPPH regulates cell proliferation and cell cycle via modulating MDM2/p53 signaling in ESCC [33]. AWPPH acted as an oncogene to interact with YBX1 to activate the expression of SNAIL1 and PI3K/AKT pathway in the HCC [11]. Wnt/β-catenin signal pathway involved in the regulation of cell proliferation, migration and invasion in certain cancers [34,35], and AWPPH could promote the proliferation, migration and invasion of BC, OC and NSCLC by activating this pathway [12,30,36]. Several important pathways were also conformed to be modulated by AWPPH in cancers, including MDM2-p53 pathway esophageal squamous cell carcinoma [33] and MEK/ERK pathway in HCC [22]. Furthermore, AWPPH could inhibit colon cancer cell proliferation by down-regulating GLUT-1 [37] and mediate the metastasis and postoperative distant recurrence by up-regulating TGF-β1 [29,38,39]. Liu et al. demonstrated that AWPPH contributes to cisplatin resistance by inducing the expression of CDK1 and GTSE5, and suppressing the expression of CCNC and BIRC5. This provided a brand new insight for the cisplatin resistance of gastric cancer NSCLC [40].

Additionally, a number of studies revealed that AWPPH could act as a key competing endogenous RNA (ceRNA) or sponge for miRNAs to regulate the initiation, development, and chemoresistance of cancer. For example, in gastric cancer, Bu et al. demonstrated that AWPPH promotes cell proliferation and tumorigenesis by regulating miR-497/NTRK3 axis [24]. Recently, miR-128-3p was confirmed as a target of AWPPH in ovarian cancer by Zhu et al*.* [23]. In NSCLC, Wu et al. found that AWPPH could directly interacted with miR-204 and functioned as a ceRNA, thus regulating the expression of CDK6 [13]. Furthermore, AWPPH functioned as a ceRNA to promote malignant progression of human cancers through competitive sponging of miR-93-3p in osteosarcoma [28], miR-802 in melanoma [41], miR-206 in CRC [18], miR-1224-5p in glioblastoma [42] and miR-513a-5p in CCRCC [43].

Nevertheless, several limitations to this meta-analysis should be taken into account. First, all included studies were performed in the population from China, which may limit the applicability of our results for other ethnic population. Second, the cut-off values were lack of uniform standard in different types of cancer, which may result in some heterogeneity and affect the results of the study. Third, some of the HRs were calculated based on data extracted from the survival curves, which may not be very accurate and result in a calculation bias.

## Conclusion

To conclude, this meta-analysis revealed that AWPPH expression level served as a prognostic indicator in multiple cancers in the Chinese population. Higher expression of AWPPH was significantly associated with poorer overall survival in patients with cancers and correlated with advanced TNM stage, higher risk of LNM and MVI, and bigger tumor size. Ultimately, more high-quality studies were required to certify clinical utility of AWPPH in cancers.

## Data Availability

The data used to support the findings of this study are available from the corresponding author upon request.

## Abbreviations

AWPPH: long non-coding RNA associated with poor prognosis of hepatocellular carcinoma
BC: breast cancer
CC: cervical cancer
CCRCC: clear cell renal cell carcinoma
ceRNA: competitive endogenous RNA
CRC: colorectal adenocarcinoma
DFS: disease-free survival
DM: distant metastasis
ESCC: esophageal squamous cell carcinoma
GC: gastric cancer
HCC: hepatocellular carcinoma
LNM: lymph node metastasis
MVI: macro-vascular invasion
NOS: Newcastle–Ottawa Scale
NSCLC: non-small cell lung cancer
OC: ovarian carcinoma
ORs: odds ratios
OS: overall survival
OSCC: oral squamous cell carcinom
PC: prostate carcinoma
PFS: progression-free survival
qRT-PCR: quantitative real-time polymerase chain reaction
RFS: recurrence-free survival
TNBC: triple-negative breast cancer
